# Supplementary material for: A Qualitative Study on Young Men’s Experiences of Intentional Weight-Gain
Source: Int J Environ Res Public Health. 2023 Feb 14;20(4):3320. doi: 10.3390/ijerph20043320 (PMC9964095; doi:10.3390/ijerph20043320)
Supplement: Supplementary file 1 [file ijerph-20-03320-s001.zip › Supplementary File S1 - Baseline Topic Guide .pdf]

GlasVEGAS extension – interview topic guide - clinic – pre weight-gain

Introduction

First, I'd just like to say thank you for agreeing to take part in this interview-based study, which is an extension to the main GlasVEGAS project.

- We are University of Glasgow researchers who have worked with others - including some involved in the main GlasVEGAS study - in developing programmes to improve health and wellbeing.
- We're interested in hearing the views of a wide range of GlasVEGAS participants to understand their experiences of weight changes and of taking part in the study.
- So we're interested in men's expectations at the start, and how they feel once they have gained, and then lost weight.
- This interview at the start should take about 30 or 40 minutes – but remember that you can say you'd rather not answer a question, or stop at any time, without giving a reason.
- Provided you agree, we'll record the interview – but everything you say will be kept confidential and anonymised in all reports.

And I should also say that we're very near the start of this study – so at the moment what's included in the interviews might be a bit rough around the edges. So bear with me – and at the end I'll ask if you've any feedback or can think of important things I've forgotten to ask.

Interview topics

Just before we start, I should say that at the moment I've no idea of what you've said in any questionnaires you've completed – so I hope you'll forgive me if some of this seems a bit repetitive.

So first, can I just get a few background details? (Aiming for more contextual details.)

- Labour market position (course / occupational details). How long?
- Living situation – who? How long?
- Age

And why were you interested in taking part in GlasVEGAS?

- Personal interest?
- Good of society?
- Financial gain?

So here you are at the start of the study – and I think you've filled in a questionnaire which asked a bit about your weight and body build – so can I just ask a bit about that?

- How would you say you currently felt about your weight? Level of satisfaction?
- How about your build? Level of satisfaction?
- Are your weight or build important issues for you? Which matters most?
- Do you think men differ in how much being muscular matters? Would you say it matters for you?
- Have how you feel about weight or build or muscularity changed over time? How?

GlasVEGAS is a men-only study – do you think men and women differ in their attitudes to body shape and build?

- In what ways?
- Do you think this has changed over time?

So at the moment, would you say you had a healthy diet?

- Can you remember what you ate yesterday? (meals and snacks) Was that pretty average for you?
- Did you eat with other people?
- Did you prepare food with other people?
- Who buys your food?
- Who decides what to eat?

And what about exercise and physical activity – how active a person would you say you were?

- Describe levels last week. Was that pretty average for you?
- Did you do sports or exercise with other people?
- Who decides what you do?

So you're about to start on the weight gain part of the study. How easy or difficult do you think gaining weight will be?

- You've got to add 1,500-2,000 calories/day – have you got an idea of what that means in terms of what you'll have to eat?

And how easy or difficult do they think losing weight will be?

- So I think they've suggested the options are weightwatchers or alternate fasting days – have you thought about what might work best for you?
- Why?

Have there been other times in your life when your weight has changed?

- What happened?
- Intentional?
- What was it like?

And for this time – have you had any thoughts about how putting on a bit of weight might affect your body shape?

- Where might it show? (i.e. where fatter).

Might it affect how your body feels?

- How?

Might it affect how you feel in yourself?

- How?

And what about when you come to lose weight – have you had any thoughts about how that might affect your body shape?

Might losing the weight you've gained affect how your body feels?
- How?

Or might it affect how you feel in yourself?
- How?

Do the people round you know you're involved in the GlasVEGAS study?
Might it have any impact on them?
- The process of you gaining or losing weight – so what you actually have to do.
- Or the actual weight gain or loss – so you putting on a bit and then losing it again.
- Family?
- Friends?
- Colleagues?

Post interview

So that's all the topics from my list.
- Did I miss anything out which might be important?
- Did I ask any daft questions?
- Have you any other feedback on the interview?

And finally – you'll remember from the information sheet that our study is also hoping to talk to people after they've gained weight and once they've lost it again. We're expecting those interviews to be quite a bit shorter.
- Would you be OK with that?
- And would you have any preferences over whether it was here in the clinic or by phone?
