# Supplementary material for: A Qualitative Study on Young Men’s Experiences of Intentional Weight-Gain
Source: Int J Environ Res Public Health. 2023 Feb 14;20(4):3320. doi: 10.3390/ijerph20043320 (PMC9964095; doi:10.3390/ijerph20043320)
Supplement: Supplementary file 1 [file ijerph-20-03320-s001.zip › Supplementary File S2 - Follow-up Topic Guide.pdf]

GlasVEGAS extension – interview topic guide - clinic – post weight-gain

Introduction

First, I'd just like to say thank you for agreeing to take part in the second phase of this interview-based study, which is an extension to the main GlasVEGAS project. This interview should be slightly shorter than our previous one and should not take any longer than about 30 or 40 minutes.

So at this stage we are particularly interested in men's experiences once they have gained weight as part of the GlasVEGAS project. Please remember that you can say you'd rather not answer a question, or stop at any time, without giving a reason. Provided you agree, we'll record the interview – but everything you say will be kept confidential and anonymised in all reports.

Interview topics

So first, can you please tell me about your experience of weight gain as part of GlasVEGAS?
- What did it involve practically?

Did the actual practicalities of weight gain match your expectations?
- Was the process easier / harder?
- What was it specifically about weight gain that was easier / harder?

Was anything a big surprise? And, if so in what ways?

Did you notice particular changes in how your body looked as you gained weight?
- Any specific changes in physical appearance?
- Any changes in physical fitness/energy levels?

How did any of these changes make you feel?
- Did these feelings change over time?

Did the process (practicalities) of weight gain impact on the people around you?
Explore in relation to all three:
- Family?
- Friends?
- Colleagues?

What if at all have been their reactions to the fact you have put on some weight?
Explore in relation to all three:
- Family?
- Friends?
- Colleagues?

Have people mentioned it to you directly? If not – do you think they've noticed it?

Were these people aware of the fact you were taking part in the GlasVEGAS project and what was involved specifically?

Now that you have completed the weight gain phase of the study what are your current expectations of weight loss?
- Do you think this has changed since your experience of weight gain?
- In what ways?

How easy or difficult do you think losing weight will be?
- Why? Probe: Confident / apprehensive
- What about some of the habits you might have formed – do you think it will be easier / harder than expected to break / alter these while trying to lose weight?

The suggested options are weightwatchers or alternate fasting days – have you thought about what might work best for you? Has this changed since doing the weight gain part of the study?
- What about your physical activity / exercise levels?

GlasVEGAS is currently a men only study – do you think women would agree to take part in the study?
- Do you think men and women would differ in their approaches to losing weight?

How do you feel now about your decision to participate in the GlasVEGAS project?

During the study you will have experienced a wide range of objective physical measurements – could you tell me what it was like having some of these measurements done and receiving any information on these?
- Did having these measurements carried out make you feel differently about yourself? And, if so in what ways?

Post interview

So that's all the topics from my list.
- Did I miss anything out which might be important?
- Did I ask any daft questions?
- Have you any other feedback on the interview?

And finally – you'll remember from the information sheet that our study is also hoping to talk to people once they've lost weight again. We're expecting those interviews to be of similar duration to this interview.
- Would you be OK with that?
- And would you have any preferences over whether it was here in the clinic or by phone?
